# Underlying Dimensions of Social Cohesion in a Rural Community Affected by Wartime Violence in Colombia

**DOI:** 10.3390/ijerph16020195

**Published:** 2019-01-11

**Authors:** Ignacio Ramos-Vidal, Ilse Villamil, Alicia Uribe

**Affiliations:** 1 Departamento de Psicología Social, Facultad de Psicología, Universidad de Sevilla, Calle Ramón y Cajal s/n, 41018 Sevilla, Spain; 2 Grupo de Investigación CAVIDA, Escuela de Ciencias Sociales y Humanas, Universidad Pontificia Bolivariana, calle 63#6-58, Monteria 230017, Colombia; ilse.villamil@upb.edu.co (I.V.); alicia.uribe@upb.edu.co (A.U.)

**Keywords:** social cohesion, war, homophily, community participation, personal networks, community leadership, sense of community

## Abstract

War deteriorates the quality of life of the population and profoundly alters social dynamics. We discuss a rural community in northern Colombia whose population was the victim of a massacre and examine the main components that model social cohesion: (a) positive attitudes towards the community, (b) prosocial behaviours and (c) interpersonal relationships. This investigation is a cross-sectional empirical study that includes an analysis of social support networks. The research involved 106 residents, (81.1%, women), with an average age of 42.5 years (standard deviation (SD) = 16.4), who have lived in the community an average 28.8 years (SD = 18.75). Cluster analysis shows that there are two types of personal networks based on homophily and the duration of the ego-alter relationship. The networks that provide the most types of social support shows a moderate level of homophily according to the type of relationship and place of origin and in which the duration of the ego-alter relationship is shorter, compared to networks characterized by high homophily and in which the duration of the ego-alter relationship is longer (*χ*^2^ = 5.609, *p* < 0.018). Homophily based on place of residence actively affects the sense of community and social cohesion. Moreover, the sense of community is the variable that most affects social cohesion (β = 0.650; *p* < 0.001) and is, in turn, determined by prosocial behaviour (β = 0.267; *p* < 0.006). However, prosocial behaviours do not significantly affect interpersonal relationships or community cohesion. The results are discussed to promote social development strategies aimed at building individual, organizational and community capacity to foster psychosocial well-being in post-war contexts.

## 1. Introduction

### 1.1. War and Psychosocial Processes

War produces remarkable effects in social dynamics during the time in which the conflict is active and in the post-conflict stage [1]. Different studies show that countries that have suffered wars experience notable changes in economic, political and social systems. These investigations usually focus on showing the consequences of conflict in times of war, but on rare occasions the psychosocial processes that trigger these transformations are examined. It is also rare to identify studies that analyse the transition from when the conflict is active until the post-conflict phase begins. The work proposed by Wood [2] is an exception, insofar as it examines the effects that different armed conflicts in El Salvador, Peru, Sri Lanka and Sierra Leone produced in multiple phenomena, particularly in the transformations that shape the structure and composition of interpersonal links among civilians. The collateral damage from war produces abrupt changes in the structure, composition and interaction patterns of the social networks of the civilian population. Wood [2] makes a statement that summarizes this phenomenon:

“[…] *wartime polarization may reshape friendship networks in a village, fracturing the network into two distinct networks with no edge between them”.* (p. 540)

Other studies describe the transformations at the level of the country as a whole, forgetting the effects that war produces in the daily life of small communities and in the life plans of individuals [3]. This fact invites academics to study the consequences that war and episodes of violence generate in the social dynamics of local communities and to focus on conducting micro-social analysis in contexts that have notably suffered the effects of war.

During wartime, various forms of violence are exercised. Some of these forms include forced displacement, kidnapping, torture, sexual violence or massacres [4]. Such forms constitute what are known as the repertoires of violence that the conflicting sides use to achieve their objectives. Each of these modalities produces a differential effect in the population. Massacres are considered one of the maximum expressions of violence [5]. This fact makes it necessary to explain the profound impact generated by massacres, which stigmatize the community and generate serious psychosocial adjustment problems in survivors [6].

Other studies shows that the consequences of acts of mass violence such as massacres are aggravated in cases in which (a) responsibilities is not clear, (b) there is no reparation of the damage to the victims, and (c) the small size of the community is abruptly diminished [7]. Massacres produce far-reaching effects on the life of communities, as they represent a break with what came before as far as the community’s structure, composition and social dynamics [8]. Violence in its multiple manifestations produces drastic changes in (a) social cohesion, (b) the subjective perception of social justice, (c) the attachment that the population feels towards their community and (d) the degree of involvement in initiatives aimed at improving the inhabitants’ quality of life. 

### 1.2. Fundamentals of Social Cohesion

Classical studies describe cohesion as the set of social forces that encourage individuals to remain in a community [9]. These initial definitions consider that there are certain factors of attraction and expulsion that condition the desire to belong to social structures. More recent studies identify social cohesion as a latent construct composed of multiple variables [10]. Evidence suggests that groups reach optimal levels of cohesion when conditions at the group level promote in members positive attitudes and behaviours related to belonging to the group and when the said cohesion is based on high-intensity interpersonal relationships [11]. Social cohesion connects the social micro, meso and macro levels simultaneously. A rigorous analysis of social cohesion requires evaluating (a) the attitudes that members experience towards the community, (b) the observed behaviours that denote prosocial and altruistic behaviours, and (c) the interpersonal links that connect the members.

Attitudes within the social cohesion approach refer to the will to continue forming part of the group, to the sense of belonging and the degree of identification that individuals experience with respect to the group. Prosocial behaviours reflect the specific behaviours of the group members in terms of the level of involvement in decision-making processes, the assumption of leadership roles, and participation in activities of common interest.

In relation to attitudes, the sense of belonging is one of the antecedents that perhaps best explains from a subjective level the decision to continue belonging to communities. The psychological sense of community (PSoC) [12] assesses dimensions of PSoC (reinforcement of needs, membership, influence and shared emotional connection) through a multidimensional model. However, some authors suggest that in addition to attitudes, it is essential to evaluate the specific behaviours that each member carries out to guarantee group cohesion. As some authors [13] point out, community participation (CP) is one of the behaviours that most affects social cohesion. CP is a prosocial behaviour based on commitment, solidarity and the norms of reciprocity that emerge in contexts of social interaction. This approach highlights the relevance of analysing specific behaviours to understand social cohesion, referring to the time and resources that members are willing to invest in order to remain united with the community.

The connection that exists between the attitudes and behaviours that determine social cohesion is adequately reflected in the synergy that exists between PSoC and CP [14]. An individual’s PSoC is a catalyst for participatory behaviours, which in turn affect other community processes such as psychological empowerment (PE) and perceived social justice [15,16,17,18]. To understand the processes that determine the level of social cohesion, it is necessary to evaluate the interaction between dimensions of attitudinal character (PSoC) and prosocial behaviours (CP and PE).

In addition to the attitudinal and behavioural aspects mentioned, researchers have paid special attention to interpersonal relationships that produce variations in the degree of social cohesion [19]. Interpersonal relationships are at the base of community cohesion [20]. Social ties shape support networks through which flow material, affective and informative resources that allow community members to meet multiple needs. These social ties shape relationships based on solidarity, reciprocity and trust, acting as a powerful mechanism that holds the community together. The structural and compositional properties of networks have frequently been used to explain the social cohesion of groups, organizations and communities.

**Figure 1:**
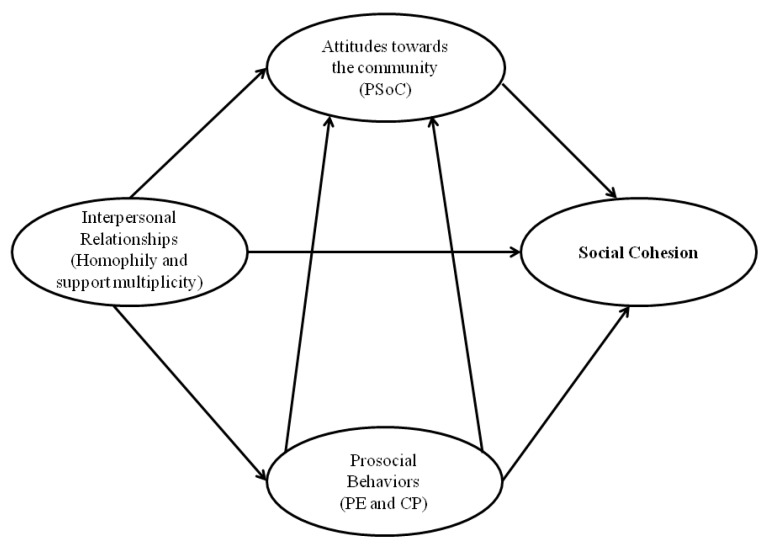
Theoretical model of the constitutive elements of social cohesion. PSoC: psychological sense of community; PE: psychological empowerment; CP: community participation.

There are multiple indicators of structure and composition of the networks that are used to analyse group cohesion. Among these indicators are (a) density, (b) the intensity of these relationships, (c) the indicators that analyse the processes of selection and influence according to certain attributes of the actors (i.e., homophily) and (d) the multiplicity of support [21,22,23,24,25]. Figure 1 illustrates the theoretical model that explains the main factors that determine social cohesion.

Considering that social cohesion is a multidimensional construct whose definition depends on the attitudes, prosocial behaviours and interpersonal relationship, it is logical to think that violent acts will produce disruptive effects on the cohesion of the communities that have suffered these types of episodes. Therefore, it is necessary to examine the factors that determine social cohesion in communities that have suffered violent acts. The objectives and the study context are detailed in the following sections.

### 1.3. Objectives

1. Analyse the structure and composition of personal networks of members of a rural area affected by violence.
2. Determine the effect that interpersonal relations, attitudinal factors (PSoC) and behavioural factors (CP and PE) produce in community cohesion. For this purpose, nine regression models (linear and multiple) were performed. Five regressions are developed to identify the associations between the independent variables (interpersonal relationships (homophily and support multiplicity), attitudes towards the community (PSoC) and prosocial behaviors (PE and CP)). While the rest of the models are proposed to examine the relationships between the independent variables and social cohesion.

### 1.4. Study Context

The present study was conducted in a rural area located in the northern zone of Colombia, in the so-called Caribbean region. This zone has been one of the most punished by paramilitary groups since the early 1980s. Until the early 1990s, the rural areas suffered the consequences of the struggle between the Revolutionary Armed Forces of Colombia (FARC) and paramilitary groups for the control of strategically located *latifundios* (large-scale rural farms) and drug-trafficking routes [26]. This history, together with institutions’ lack of attention to the demands of the civilian population, has historically affected the low rates of social development in the region, especially in rural communities [27].

Since 1985, in the Department of Córdoba alone, there have been more than 240,000 victims registered in the Unified Register of Victims (Registro Único de Víctimas, RUV), of whom approximately 40,000 correspond to severe violent acts (forced disappearance and homicide). This research was carried out in a rural area belonging to the municipality of Buenavista. This municipality has 21,628 inhabitants, of whom 61.6% live in rural areas according to the last official census of 2015. In this municipality, there are 3094 registered victims, which means that approximately 14.03% of the population are victims recognized by the state. Of the total of registered victims, 753 correspond to forced disappearances and homicides. 

The context of this study was selected because in 1988 the first massacre attributed to the Colombian paramilitaries took place in the community under study. According to official data, 27 inhabitants of the community died. It has been documented that this type of event seriously undermines social cohesion, and in this concrete event, the three factors that, according to Hoover [7], aggravate the consequences of violence coincide: (a) impunity, (b) failure to repair the damage, and (c) significant reduction in the size of the community.

## 2. Materials and Methods 

### 2.1. Participants and Sampling

The eligibility criteria were to be over 18 years of age and to have lived stably in the community. A total of 106 residents participated, most of them women (81.1%), with an average age of 42.5 years (SD = 16.4) who have a basic level of education (72.4%) and who work in domestic work (68.9%) and in agriculture or livestock (11.3%). The participants have lived for an average of 28.8 years in the community (SD = 18.75). We decided to use snowball sampling to identify potential participants due to the villagers’ reluctance to participate in the study. Each participant received a family food bonus equivalent to $25. Although there is no official census, it is estimated that the current size of the community is approximately 250 people. Assuming the veracity of this data, the estimated coverage would be 42.4% of the community. All procedures performed in studies involving human participants were in accordance with the ethical standards of the Universidad Pontificia Bolivariana and with the 1964 Helsinki declaration and its later amendments or comparable ethical standards.

### 2.2. Instruments

#### 2.2.1. Independent Variables (Attitudinal and Behavioural)

To evaluate PSoC (attitudinal component), we used the second version of the Sense of Community Index (SCI-II) [28]. The scale evaluates the four dimensions of the original theoretical model. The dimensions evaluated are membership, influence, meeting needs and shared emotional connection. The SCI-II consists of 24 items rated on a Likert scale from 1 to 4, where 1 is not in agreement and 4 completely agree (Example of item, *“being a member of this community makes me feel good”*). The scale presents a factorial structure congruent with the multidimensional model of McMillan and Chavis [12] and has optimal psychometric properties (α = 0.83). The average score of the complete scale is 3.07 (SD = 0.4), which would reflect a moderately high PSoC.

To measure CP, we use the scale proposed by Speer and Peterson [29]. The instrument consists of five items that evaluate the frequency with which different participatory behaviours are developed (example of item, *“How often do you attend meetings to obtain information about social services or resources available in your community?”*). The instrument has adequate psychometric properties (α = 0.88). The average score of the complete scale is 2.6 (SD = 1.06). These data indicate that the respondents occasionally participate in prosocial activities.

The evaluation of PE was carried out with a scale that measures the leadership factor as an essential part of empowerment [30]. The instrument consists of five items that are assessed on a four-point Likert scale, where 1 is equal to no agreement and 4 to complete agreement (Example of item, *“I am often the leader in the groups of which I am part”*). The scale presents acceptable reliability (α = 0.79). The average score of the complete scale is 2.3 (SD = 0.79), reflecting a modest level of empowerment linked to leadership roles.

For this study, we designed an instrument to assess the extent to which participants consider that the massacre changed interpersonal relationships and social dynamics (e.g., by restricting the celebration of public events). The scale consists of seven items that examine the impact of the massacre on each of the dimensions for which it is asked. The participants responded on a scale of 1 to 4, where 1 means that the massacre did not produce any impact and 4 means that it had a great impact (Example of item, “*What effect did the massacre have on the number and intensity of relationships that you maintain with other residents of your community?*”). The scale presents acceptable psychometric properties (α = 0.89). The average score of the complete scale is 3.04 (SD = 0.87), indicating that for the participants, the massacre caused a notable impact on interpersonal relationships and social dynamics.

Each participant completed a questionnaire to identify the members of their social support network. The design of the instrument was inspired by the work of Barrera [31], in which the author proposes different questions (name generators) in which he asks about the types of social support that each member of the network (alter) provides to the person interviewed (ego). An example of a name generator used is “*With whom do you turn to for private, intimate or very personal matters?*”. We established the limit of the network at 20 alters. Several studies show that limiting the size of the personal network to 20 actors makes it possible to capture the compositional diversity and the social circles that model personal networks [21,32]. Once the components of the personal network have been identified, the interviewee must indicate the relationships maintained among the 20 alters to identify the complete structure of the personal network. The resulting information was transferred to an adjacency matrix, and the indicators that we describe below were calculated with the UCINET software (version 6.664, Analytic Technologies, Harvard, MA, USA) [33]. Each participant offered the following information for each nominated alter: (1) sex, (2) age, (3) type of relationship (e.g., kinship, friendship, neighbour, etc.), (4) place of residence and (5) multiplicity of social support (types of social support provided to ego).

The indicators used to evaluate interaction patterns in personal networks are the multiplicity of social support and homophily based on the place of residence. Support multiplicity describes the different types of social support (material, informative and affective) that the alters provide to the ego. This measure is computed by adding the six types of support that each member of the personal network provides to the ego and dividing that sum by the number of members of the network. Homophily examines the extent to which actors tend to establish relationships with other actors with whom they have attributes in common [23]. To describe the patterns of interaction in personal networks, we use homophily based on sex, type of relationship and place of residence. To determine the effects of homophily on the attitudinal and behavioural components and on the cohesion of the community, we use homophily according to the place of residence. The values of homophily range between −1 (pure homophily) and +1 (pure heterophily). Values close to −1 mean that the members of the personal network tend to establish relationships only with other subjects who reside in the same community, while values closer to +1 indicate that the components of the ego network tend to prefer to establish relationships with people who reside in other communities.

#### 2.2.2. Dependent Variable

The measurement of community cohesion was carried out with an instrument developed by Moncada and colleagues [34] and adapted for the purposes of the study. The instrument has nine items that are evaluated on a Likert scale from 1 to 4, where 1 is equal to no agreement and 4 to complete agreement (example of item, *“Everyone in this community participates in decision making about policies and actions to follow”*). The instrument has acceptable psychometric properties (α = 0.74). The average score of the complete scale is 3.2 (SD = 0.45), indicating that the participants perceive a high level of social cohesion.

### 2.3. Analysis of Data

To characterize the personal networks of the participants (Objective 1), visual representation techniques were used, examining the structural properties and attributes of the alters. To establish a classification of personal networks based on cohesion indicators, we developed a cluster analysis [35]. To examine the covariance relationships between the independent and dependent variables (Objective 2), several simple and multiple linear regression analyses were performed. 

## 3. Results

### 3.1. Characterization of Personal Networks (Objective 1)

The personal networks of the interviewees have a high degree of density and a general homophilic tendency, based on the different attributes. Table 1 presents the main structural and compositional indicators of the networks.

Structural cohesion measures show high values. The average density of personal networks is 96%, which is a value above that of this indicator in social support networks [21]. The networks have a high transitivity, which suggests that practically all alters know each other, with a variable intensity in the strength of the tie. In the opposite direction, the structure is characterized by a low centralization, suggesting a similar pattern of connections between all the alters. The analysis of the subgroups shows that, on average, the networks are composed of a single highly cohesive component, showing a high degree of overlap. This fact reflects that the alters share the same social circles.

Studies show that in a variable measure, the overlap is usually caused by the alter attributes [36]. If we examine the indicators of homophily, we observe a moderate homophilic tendency according to sex (−0.1) and type of relationship (−0.16) and a pronounced homophilic tendency according to the place of residence (−0.52). In this case, (a) the geographical isolation of the community, (b) the lifestyle of this type of rural population, and (c) the antecedents of this zone facilitate and reinforce the intracommunity social bonds, influencing the strengthening of highly cohesive interpersonal relationships [37].

To determine the composition of personal networks, we analyse the attributes of the alters. It is observed that approximately half of the alters are family (49.9%), the second most-represented group are friends (28.9%), the third are neighbours (16.6%) and, finally, acquaintances represent a small group (2.8%). Depending on the place of residence, we observed that 81.4% of the alters reside in the same community as the ego. Finally, we asked each participant to indicate the duration of the relationship he or she maintains with each alter. On average, the participants have known each alter for 23 years (SD = 10.15). Taking into account the average age of the participants (42.5%) and the time they have lived in the community (M (mean) = 28.8 years), we can affirm that the networks present a high degree of stability. This fact may be due to the low dynamism regarding lack of socialization opportunities and the difficulties of accessing other social groups. Several studies suggest that having options for socializing is the main trigger for changes in the composition of personal networks [38]. We developed a cluster analysis in order to categorize personal networks based on structural parameters and alter attributes. Table 2 shows the results of the cluster analysis.

Cluster analysis identifies two types of networks based on (a) homophily based on the type of relationship, (b) homophily based on the place of residence of the alter and (c) the duration of the ego-alter relationship. We decided not to include density as grouping variable, because most networks have cohesion indexes close to 100%, so these variables do not allow typologies to be established. The networks of the first cluster (*n* = 43) present a pronounced homophilic tendency according to the type of relationship and place of residence, and they are very stable networks considering the average length of the ego-alter relationship. On the other hand, in the networks that make up the second cluster (*n* = 62), there is no homophilic tendency based on the type of relationship, they show a moderate homophilic tendency according to the place of residence, and they are comparatively less stable if we look at the duration of the ego-alter relationship.

To illustrate the two types of personal networks identified, we proceeded to visualize two representative networks of two groups. The colour of the nodes represents the membership of the alter (the residents of the community in which the study was carried out are represented in grey, and the residents of other populations are represented in white); the size of the node represents the duration of the ego-alter relationship (larger size represents longer duration of the relationship); the shape of the node indicates the type of ego-alter relationship (circle = relatives, square = friends, triangle = neighbours); and finally, the thickness of the tie represents the intensity of the relationship (thin ties represent people who know each other casually, and thick ties represent people who know each other well). 

**Figure 2:**
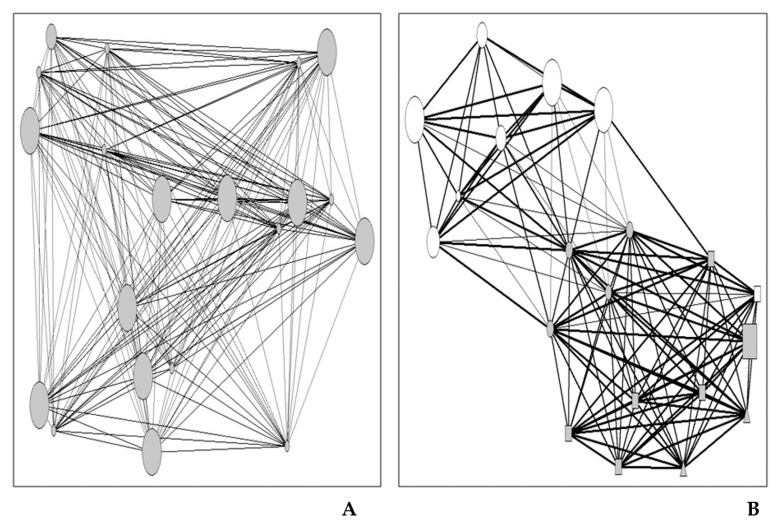
(**A**) Personal network belonging to cluster 1 (Case 29) and (**B**) personal network belonging to cluster 2 (Case 89).

The first network (Figure 2A) belongs to a 36-year-old woman who has lived in the community her entire life. The second network (Figure 2B) corresponds to a 26-year-old woman who, like the previous woman, has always lived in the same population. Despite having this point in common, both networks show notable differences. The networks of cluster 1 are more homogeneous and are composed mostly of family members residing in the same community. The networks of the second cluster include people living in other populations and others who are not part of the family circle. Figure 2B shows that the network consists of two cohesive subgroups: the largest cluster comprises family members and friends residing in the same community, while the second subgroup includes family members residing in other communities. This second network describes the relational context of a person who distributes her social resources in two differentiated interaction spaces, although they present a certain degree of overlap. This type of relational context facilitates access to other groups, a factor that increases the opportunities for raising one’s status in the social structure [39].

We applied the Kruskal–Wallis test to identify the differences between the networks of both clusters based on the multiplicity of social support. In the networks of the second cluster, the alters provide more types of social support in comparison with the alters of the networks of the first cluster (*χ*^2^ = 5,609; Degrees of freedom (*df*) = 1; *p* < 0.018). These results suggest that the patterns of interaction between the alter (i.e., homophily based on the place of origin and the type of relationship) and the duration of the ego-alter relationship determine the provision of social support received by ego. Additionally, we examine whether there are differences between the two clusters based on the massacre’s impact on interpersonal links and on social dynamics, yielding non-significant results (*χ*^2^ = 0.781; *df* = 1; *p* < 0.377).

### 3.2. The Incidence of Interpersonal Relationships, Attitudinal Factors (Psychological Sense of Community, PSoC) and Behavioural Factors (Community Participation, CP, and Psychological Empowerment, PE) in the Social Cohesion of the Community (Objective 2)

We develop regression analyses in order to identify covariance relationships between the dimensions that determine social cohesion. First, the correlation matrix between the variables analysed is shown in Table 3, and in a second step, the results of the regression models are presented using the same illustrative figure shown in the introduction.

The correlation matrix shows a strong association between the sub-dimensions of PSoC, which in turn present strong correlations with the dependent variable. The dimensions of the attitudinal component (PSoC), with the exception of the emotional connection, maintain a significant association with the PE variable. However, significant covariance relationships between the PSoC and participatory behaviours are not identified. There is also a strong association between the PSoC satisfaction of needs dimension and homophily based on place of residence, not showing covariations with the multiplicity of social support.

The behavioural components (CP and PE) do not present significant correlations with social cohesion, although they maintain a strong association with one another. However, CP does not present covariance relationships with the rest of the variables analysed. PE shows a modest association with the dimensions of membership and influence and also with the complete scale of PSoC. However, it does not present significant covariations with respect to interpersonal relationships (homophily and support multiplicity).

Homophily based on the place of residence shows significant correlations with the dependent variable, with the PSoC satisfaction of needs dimension, with the complete PSoC scale and with the multiplicity of social support. This variable does not present covariance relationships with the behavioural components evaluated. 

**Figure 3:**
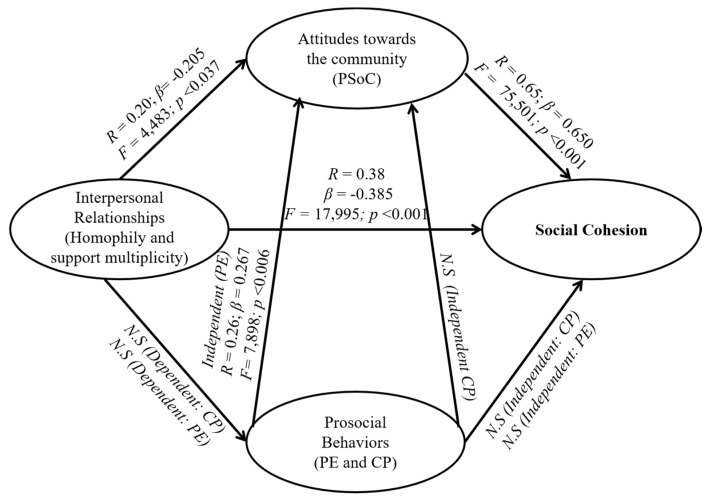
Theoretical model of the constitutive elements of social cohesion indicating the results of the regression coefficients.

Figure 3 describes the complex relationship maintained by the attitudinal, behavioural and relational components with social cohesion. PSoC is the factor that individually has a more direct effect on social cohesion, explaining 65% of the variance of the dependent variable. Interpersonal relationships, and in particular, homophily based on the place of residence, have a modest effect on (positive) attitudes towards the community, but this variable does not determine prosocial behaviours. However, the homophilic tendency displays a consistent effect on social cohesion, suggesting that the dynamics of interaction based on this process are key to activating community cohesion. 

Ultimately, we observe that prosocial behaviours, particularly CP, are detached from attitudes and interpersonal relationships and do not seem to affect social cohesion in any way. However, there are two aspects that we must highlight. First, CP and PE are mutually determined, suggesting that involvement in prosocial initiatives is a preferred way to increase self-determination and control over one’s surroundings [16,40]. Second, community leadership (PE) behaviours tend to have positive effects on attitudes towards the community and vice versa. This second finding shows that prosocial behaviours mediate perceptions and attitudes towards the community.

## 4. Discussion

War and, in particular, certain violent episodes such as massacres, can alter the social cohesion of the communities that suffer this phenomenon, even decades after the event [1,2,3,5]. This article explores the components of social cohesion and the factors that define it, analysing for this purpose a rural community in northern Colombia that witnessed a massacre three decades ago. We apply a theoretical model that shows that social cohesion is based on the attitudes, prosocial behaviours and interpersonal relationships that connect the members of a community.

There are few studies that use structural analysis techniques to evaluate the personal networks of people residing in vulnerable areas who have also suffered the effects of war. The results show that the members of the community have very dense, homogeneous and highly homophilic personal networks. This type of structural configuration can limit access to information and resources that improve the quality of life of the population. This phenomenon occurs because psychosocial processes that can constrain collective action emerge in groups and highly cohesive personal networks [41,42]. However, in this particular case, it seems that (a) the composition of the networks and their high levels of (b) cohesion and (c) homophily are due to more than the premeditated decisions, habits and lifestyle that characterizes the inhabitants of rural communities such as those analysed in this work. Different studies show that the geographical isolation and the small community size produce a high degree of homogeneity in the composition of personal networks, simultaneously affecting the high density indexes and the overlap in the provision of social support [43]. These factors provide stability to intracommunity relations, but at the same time they limit the options for achieving greater levels of social development by constraining access to diverse social groups.

Social cohesion is also based on the degree of uniformity that exists in positive attitudes towards the community, in prosocial behaviours and in the interpersonal ties that connect the members of the group. This nature implies that in addition to examining the interaction between the components of social cohesion, for such cohesion to crystallize and increase collective efficacy there must be consensus in the community members’ evaluations of these factors [44]. In the evaluated community there is a high uniformity in positive attitudes towards the community as well as in some properties of personal networks such as density and homogeneity. This level of agreement can encourage community cohesion. However, there are differences in other properties of interpersonal relationships, particularly in the indexes of homophily and in the multiplicity of social support, that can inhibit social cohesion. The moderate levels observed in prosocial behaviours also make it difficult for the community to take advantage of the potential that social cohesion offers in promoting subjective well-being and quality of life [45].

The analysed community was the victim of a massacre, considered one of the most severe forms of collective violence [5]. In this research, we start from this base and consider that this episode of violence triggered multiple psychosocial processes marked by fear, loss of trust and weakening of inter-community relationships that produced immediate changes in the social dynamics and lifestyle of the population. This phenomenon may be the cause of the structural configuration of personal networks, particularly with regard to their high density and homogeneity. Faced with fear and loss of social capital, it is likely that the members of the community replicated their relational context by concentrating interpersonal relationships in the nuclear family residing in the same locality, significantly reducing inter-community ties. Considering the theoretical connection that exists between interpersonal relationships and the other components that model social cohesion, it is feasible to suppose that the changes that the massacre caused in personal networks in turn caused substantive changes in attitudes towards the community and in prosocial behaviours.

The analysis of personal networks allows us to differentiate two profiles according to the patterns of interaction and the composition of personal networks. Networks that exhibit high homophily and high stability based on the duration of the ego-alter relationship (cluster 1) provide fewer types of social support than networks that are less stable and in which homophily is less pronounced. This finding is paradoxical if we assume that the evidence shows that stable, homogeneous and highly cohesive networks tend to be more effective in providing social support. However, it is likely that in this community, the structural properties mentioned produce differential effects. When the possibilities of establishing contacts with other communities are limited due to isolation, geographic dispersion and the weakening of inter-community relations, the positive effects of density and homogeneity can be diluted over the passage of time. This effect is produced by two factors adequately explained by the network theory. On the one hand, the duration of the relationship and the concentration of relationships within the same community can lead to the “saturation” of the sources of social support, that is, in relational terms they may lead to a depletion of support providers. On the other hand, the endogamy and homogeneity that characterize the networks cause interaction patterns to emerge characterized by high homophily indexes that produce a high degree of relational overlap. This function may limit access to positive interpersonal relationships beyond the boundaries of the community.

The lack of accessibility to other people who could provide other varieties of social resources can affect the high degree of saturation and homogeneity that characterizes the participants’ networks [46]. At the same time, the constriction of networks and geographic isolation make it difficult for community members to establish non-redundant daily contacts with people who occupy higher positions in the social structure. This fact is of special relevance considering that recent studies show that access to this type of people is crucial to improve living conditions and climb the social ladder [39].

This work is not without limitations. In the first place, the massacre that took place in the rural community analysed occurred more than three decades ago, so it is likely that other events that occurred in this period have also had an impact on social dynamics. Second, many participants were young or not born at the time of the event, so there may be a differential perception of the massacre’s effects depending on the participants’ age and whether they were direct or indirect witnesses of the event. Third, in relation to CP, in addition to evaluating the frequency of involvement in this type of activity, we recommend evaluating the impact of participatory activities. CP leads to community empowerment when positive results are obtained [18]. However, the antecedents also show that when the CP results do not meet the expectations of those who participate, the reverse occurs, producing losses of control over the environment that lead to alienation and the generation of negative attitudes towards the community. Finally, we consider it necessary to combine the use of name generators to identify the components of the personal networks of the participants. We believe that it is advisable to use other procedures such as the elicitation of daily contacts or the use of ethnographic observation techniques in order to capture all the richness of the social fabric of the members of the community.

## 5. Conclusions

Although war has devastating effects on community cohesion and its attitudinal, behavioural and relational components, there are strategies capable of rebuilding the social environment, promote a shared positive vision about social justice and improving the quality of life of the population. Some of these strategies require establishing mechanisms that reduce the perceived costs for community members to decide to get involved in community development initiatives. Indeed some proposals suggest that prosocial and altruistic behaviour may be triggered as a consequence of negative experiences that have place after violent episodes [47]. Community participation, the assumption of leadership roles in the local context and being part of associative movements promote community strengthening [14,15,16]. Through involvement in prosocial activities, community members can have better control over their lives and increase their autonomy, while these activities facilitate the establishment of interpersonal links that are the basis for strengthening the social cohesion and the quality of life of communities that have suffered violent episodes. 

To increase social cohesion, it is necessary to generate the necessary conditions for people to acquire control over their environment and achieve self-determination [48]. In the community under study, it is essential to build organizational capacity that makes it possible to create mediating institutions, such as associations and community-based organizations capable of (a) articulating collective action, (b) establishing hierarchy in social demands, and (c) designing and implementing intervention programmes. 

Finally, some key findings are highlighted with the objective to better evaluate and intervene in communities affected by wartime violence:

1. Understand the degree of cohesiveness within the communities is a crucial point to design and implement intervention programs adapted to the community structure.
2. For improving the perceived well-being and quality of life in communities that have suffered severe violence episodes, it is essential to increase the connections of individuals with their social environment (i.e., membership), in turn activating prosocial behaviours (i.e., civic engagement) which are antecedents to promoting the acquisition of control over their lives (i.e., empowerment).
3. Personal networks analysis is a promising method for capturing the individuals’ relational context. The structure and composition of personal networks offer valuables clues to identify the degree of integration of subjects in their communities.
4. Homogeneous and highly cohesive personal networks provide bonding social capital and offer multiple kinds of social support to ego, but constrain the opportunities to establish inter-community relationships with heterogeneous groups (bridging social capital) which are basic to overcoming situations of vulnerability.

## Figures and Tables

**Table 1:** Structural indicators of personal networks (*n* = 106).

Indicator	Minimum	Maximum	Mean (M)	SD
Density	0.33	1	0.96	0.11
Number of ties	126	380	366.6	37.35
Transitivity	21.62	100	95.12	12.57
Centralization	0	70.36	3.48	9.86
Number of subgroups	1	5	1.31	0.70
Multiplicity of social support	0	4	1.4	0.82
Homophily depending on the place of residence	−1	0.55	−0.52	0.50
Homophily according to sex	−1	0.17	−0.1	0.22
Homophily depending on the type of relationship	−1	0.55	−0.16	0.42
Average length of ego-alter relationship (years)	1	48.25	23.03	10.15

**Table 2:** Clusters and final cluster centres of structural and compositional indicators of personal networks.

Variable	Cluster 1 (*n* = 43)	Cluster 2 (*n* = 62)
	Final Centres	Final Centres
Homophily based on the type of relationship	−0.31	−0.06
Homophily based on the place of residence	−0.59	−0.49
Duration of the ego-alter relationship	33.15	16.03

Note: the procedure has converged in two iterations.

**Table 3:** Matrix of bivariate correlations between the variables.

Variables	1	2	3	4	5	6	7	8	9	10
1. Social cohesion										
2. Satisfaction (PSoC)	0.52 *									
3. Membership (PSoC)	0.48 *	0.47 *								
4. Influence (PSoC)	0.47 *	0.41 *	0.54 *							
5. Connection (PSoC)	0.58 *	0.38 *	0.53 *	0.55 *						
6. Complete PSoC	0.65 *	0.72 *	0.79 *	0.82 *	0.78 *					
7. PE	0.03	0.01	0.24 **	0.35 *	0.17	0.26 *				
8. CP	−0.01	0.04	0.05	0.16	0.01	0.09	0.41 *			
9. Residence homophily	−0.38 *	−0.33 *	−0.07	−0.09	−0.15	−21 *	0.01	−0.10		
10. Multiplicity	−0.12	−0.02	−0.02	−0.10	−0.10	−0.03	0.06	0.08	−0.32 *	

Note: * *p* < 0.001; ** *p* < 0.05. PSoC: psychological sense of community; PE: psychological empowerment; CP: community participation.
